# Platycoside N: A New Oleanane-Type Triterpenoid Saponin from the Roots of *Platycodon grandiflorum*

**DOI:** 10.3390/molecules15128702

**Published:** 2010-11-30

**Authors:** Wei Li, Wei Zhang, Lan Xiang, Zi Wang, Yi-Nan Zheng, Ying-Ping Wang, Jing Zhang, Li Chen

**Affiliations:** 1 College of Chinese Material Medicine, Jilin Agricultural University, Changchun 130118, China; E-Mail: liwei7727@126.com (W.L.); 2 School of Pharmacy, Shangdong University, Jinan 250012, China; 3 Institute of Special Wild Economic Animals and Plant, CAAS, Jilin 132109, China; 4 Norman Bethune College of Medicine, Jilin University, Changchun 130021, China

**Keywords:** platycoside N, *Platycodon grandiflorum*, triterpenoid saponin

## Abstract

A new oleanane-type triterpenoid saponin, named platycoside N (1), together with six known saponins, was isolated from the roots of *Platycodon grandiflorum*. On the basis of acid hydrolysis, comprehensive spectroscopic data analyses and comparison with the spectral data of the known compounds, its structure was elucidated as 3-*O*-β-D-glucopyranosyl-(1→6)-β-D-glucopyranosyl-2β,3β,16α,23-tetrahydroxyolean-12-en-28-oic acid 28-*O*-β-L-rhamnopyranosyl-(1→2)-α-L-arabinopyranoside. The six known compounds were platycodin D (2), deapioplatycodin D (3), platycodin D_3_ (4), deapio- platycodin D_3_ (5), platycoside E (6) and deapioplatycoside E (7).

## 1. Introduction

**Figure 1:**
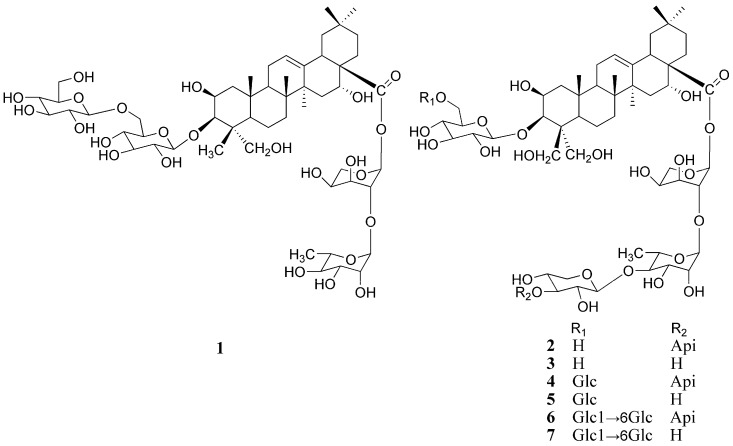
Chemical structures of compounds **1-7**.

The roots of *Platycodon grandiflorum* A. DC (Campanulaceae), Platycodi Radix, are traditionally used as food and a herbal medicine in the treatment of a wide range of diseases, including bronchial asthma, hepatic fibrosis, bone disorders [1,2,3,4], hypercholesterolemia and hyperlipidemia [5]. The principal bioactive constituents of this herb are triterpenoid saponins (platycosides), which exhibit a variety of pharmacological activities, such as anti-inflammatory [6,7], anti-obesity [8,9,10,11], anti-cancer [12,13,14,15] and hypoglycemic effects [16,17]. To date, more than 30 saponins have been isolated from this plant [18,19,20,21,22,23,24,25]. In order to find more bioactive compounds, we have now studied the chemical constituents of *P. platycodiflorum*, and in this paper, we report the presence in this species of a new oleanane-type triterpenoid saponin, named platycoside N, together with six known compounds, from the roots of *Platycodon grandiflorum*. (Figure 1)

## 2. Results and Discussion

**Figure 2:**
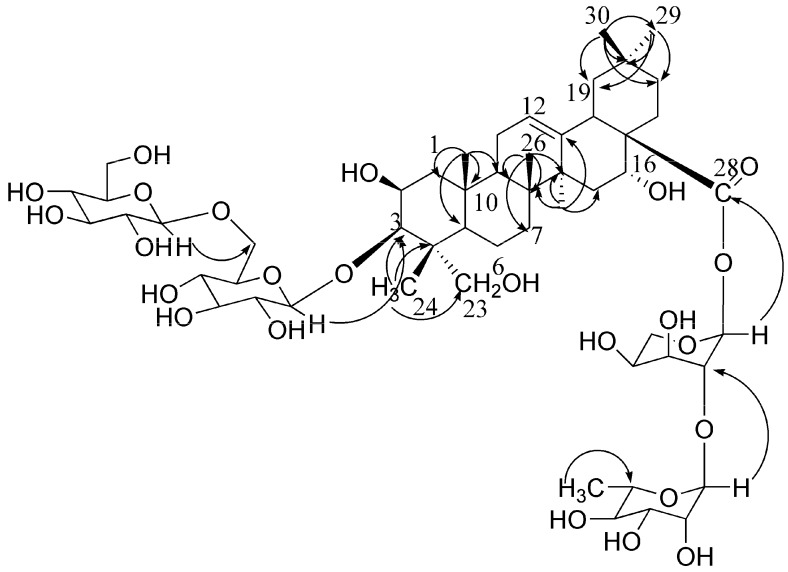
The key HMBC correlations of compound 1 (from H to C).

Platycoside N (**1**) was a white amorphous powder, and its molecular formula C_53_H_86_O_24_ was determined based on the HR-ESI-MS spectra. The oleanane-type triterpenoid saponin nature of compound **1** was revealed through analysis of its spectral features. The IR spectrum exhibited absorptions at 3,425 cm^−1^ (OH), 1,647 cm^−1^ (ester carbonyl), and 1,616 cm^−1^ (double bond). Six methyl groups (δ 0.89 × 2, 0.98, 1.17, and 1.58 × 2) and one olefinic proton (δ 5.46, br s) of the aglycon were observed in the ^1^H-NMR spectrum. The ^13^C-NMR spectrum showed that the aglycon had six methyl carbons at δ 16.0, 18.6, 17.7, 27.2, 33.3, and 24.8, two olefinic carbons at δ 123.1 (CH) and 144.5 (C), one oxymethylene and three oxymethine carbons at δ 66.6, and 70.1, 74.2 and 83.1, respectively, and one carbonyl carbon at δ 176.0 (Table 1). The information of the ^1^H-NMR spectrum coupled with the ^13^C-NMR spectrum indicated that **1** had 2β,3β,16α,23-tetrahydroxyolean-12-en-28-oic acid (polygalacic acid) as an aglycon [20]. The ^13^C-NMR spectrum showed 53 signals, of which 30 were assigned to a triterpenoid moiety and 23 to the saccharide portion. The downfield shift of C-3 (δ 83.1) and for the upfield shift of C-28 (δ 176.0), revealed that the sugar moieties were attached to the aglycon at these two positions. The ^1^H and ^13^C-NMR spectra of **1** exhibited four anomeric protons at δ 5.10 × 2 (2H, d, *J* = 7.5 Hz), 6.27(1H, d, *J* = 2.5 Hz), 5.68 (1H, br s) ppm and carbons at δ 106.7 × 2, 93.9, 101.5 (Table 1). In the ^1^H-NMR spectrum, one methyl signal at δ 1.59 (3H, d, *J* = 5.5 Hz) belonging to rhamnose was observed. In addition, the monosaccharides were identified as glucose, rhamnose and arabinose by TLC and a combination of DEPT, HMQC and HMBC experiments. Acid hydrolysis of **1** also gave glucose, arabinose and rhamnose in a ratio of 2:1:1 respectively, as confirmed by GC analysis of the respective trimethylsilyl derivatives [20]. The ^1^H- and ^13^C-NMR and 2D-NMR analysis indicated that all the monosaccharides of **1** were in pyranose forms. The β-anomeric configurations of the D-gulucose units were determined by its ^3^*J*_H1,H2_ coupling constants (7.5 Hz). The α-anomeric configurations of the L-arabinose and L-rhamnose were determined by the broad singlet of their anomeric protons [24]. The linkages between sugar moieties and C-3 of the aglycon were corroborated through HMBC experiments, *i.e.*, H-1 (δ 5.10) of the terminal glucose correlated with C-6 (δ 70.8) of the inner glucose, and H-1 (δ 5.10) of the inner glucose correlated with C-3 (δ 83.1) of the sapogenin. The linkages of sugar moieties at C-28 were established based on HMBC correlations between H-1 (δ 5.68) of rhamnose and C-2 (δ 75.3) of arabinose, and H-1 (δ 6.27) of arabinose and C-28 (δ 176.0) of aglycone (Figure 2). On the basis of all the above evidence, platycoside N (**1**) was identified as 3-*O*-*β*-D-glucopyranosyl-(1→6)-*β*-D-glucopyranosyl-2β,3β,16α,23-tertahydroxyolean-12-en-28-oic acid 28-*O*-*β*-L-rhamnopyranosyl-(1→2)-*α*-L-arabinopyranoside. 

The six known saponins were identified as platycodin D (**2**), deapio platycodin D (**3**), platycodin D_3_ (**4**), deapio platycodin D_3_ (**5**), platycoside E (**6**) and deapio platycoside E (**7**) through comparison of their UV, IR, NMR and MS data with literature values [25,26].

## 3. Experimental

### 3.1. General

ESI-MS (negative mode) measurements were carried out on an Agilent 1100 series LC/MSD Trap SL mass spectrometer. HR-ESI-MS (positive and negative modes) was analyzed on a Bruker FT-ICRMS spectrometer. IR spectra were recorded on an IR-47 spectrometer. NMR spectra were recorded on a Bruker Avance DRX 400 NMR spectrometer using TMS as internal standard, and chemical shifts δ were given in ppm. Silica gel (200–300 mesh) for column chromatography and silica gel G for TLC were purchased from Qingdao Marine Chemical Factory, Qingdao, China. AB-8 macroporous resin was purchased from Tianjin Nankai factory. Preparative HPLC was performed on a Waters 600 liquid chromatography instrument with a UV detector, monitored at 210 nm using a C18 column (Zorbax Eclipse XDB, 250 mm × 9 mm; 10 μm)

### 3.2. Plant material

The roots of *P. grandiflorum* were purchased at Changchun Guangfulu market in Changchun-city of Jilin province, China and identified by Prof. Yi-Nan Zheng, College of Chinese Material Medicine, Jilin Agricultural University. A voucher specimen (No.20050116) has been deposited in the herbarium of the same college. 

### 3.3. Extraction and isolation

Dry and powdered roots of *P. grandiflorum* (2.0 Kg) were refluxed three times with 30 L of 70% methanol, 3 h each time. Extracts were concentrated, suspended in water and sequentially partitioned with ethyl acetate and *n*-butanol. The *n*-butanol fraction was subjected to macroporous resin AB-8 column and eluted sequentially with water, 30% ethanol and 70% ethanol. The 30% ethanol elution was repeatedly chromatographed on a reverse-phase column, and eluted with aqueous methanol, affording three fractions A-C. Fraction A was purified by HPLC to afford compounds **1** (23 mg), **2** (15 mg) and **3** (40 mg). Fraction B and C gave **4** (32 mg), **5** (22 mg), **6** (16 mg) and **7** (15 mg). Platycoside N (**1**): White amorphous powder; IR (KBr) cm^−1^: 3425, 2947, 1647, 1616, 1114; ESI-MS *m/z*: 1105 [M-H]^-^, HR-ESI-MS *m/z* 1105.5408 [M-H]^-^ (Calcd for C_53_H_85_O_24_, 1105.5431). ^1^H-NMR (400 MHz, pyridine-*d*_5_) δ: 0.89 × 2, 0.98, 1.17, 1.58 × 2 (each 3H, s, CH_3_ of C-26, C-29, C-30, C-24, C-25, C-27), 1.59 (3H, d, CH_3_ of rhamnose), 4.36 (1H, d, *J* = 3.0Hz, H-3), 3.82, 4.60 (each 1H, d, H-23), 4.72(1H, m, H-2), 5.10 × 2 (each 1H, d, *J* = 7.5 Hz, H-1′ and H-1of glucose), 5.68 (1H, br s, H-1 of rhamnose), 6.27 (1H, br s, H-1 of arabinose), 5.46 (1H, br s, H-12) 5.03 (1H, br s, H-16). ^13^C-NMR (100 MHz, pyridine-*d*_5_) data: see Table 1.

### 3.4. Acid hydrolysis of **1**

Compound **1** (2.0 mg) was refluxed with 4.0 M HCl (5.0 mL) for 1 h at 95 °C, and the reaction mixture was extracted with ethyl acetate. The aqueous layer was then adjusted to pH 7.0 with NaHCO_3_. After evaporating to dryness, the sugar mixture was dissolved in pyridine and developed on silica gel TLC [CHCl_3_-MeOH-H_2_O (7:3:0.5, lower phase), *n*-BuOH-AcOH-H_2_O (4:1:5, upper phase). Three spots were seen on the TLC after spraying with 4% α-naphthol-EtOH-5% H_2_SO_4_. Through comparison with authentic sugar standards (purchased from Sigma), it was found that compound **1** possessed D-glucose, L-rhamnose and L-arabinose units.

## 4. Conclusions

In summary, we have isolated a new oleanane-type triterpenoid saponin, named platycoside N (**1**), together with six known saponins from the roots of *Platycodon grandiflorum*. 

## Figures and Tables

**Table 1:** ^13^C-NMR data of compound **1** in pyridine–*d*_5_ (δ ppm).

Position	δ _C_	Position	δ _C_
1	46.8	3-O-Glc	
2	70.1	1	106.7
3	83.1	2	75.3
4	42.4	3	78.7
5	47.5	4	72.1
6	20.5	5	76.0
7	33.6	6	70.8
8	40.2	Glc	
9	47.5	1′	106.7
10	37.2	2′	74.9
11	24.4	3′	78.7
12	123.1	4′	71.6
13	144.5	5′	78.7
14	42.4	6′	62.3
15	36.2	Ara	
16	74.2	1	93.9
17	48.6	2	75.3
18	41.5	3	70.8
19	47.2	4	66.6
20	31.0	5	63.7
21	36.2	Rha	
22	32.2	1	101.5
23	66.6	2	71.6
24	16.0	3	72.7
25	18.6	4	73.1
26	17.7	5	68.7
27	27.2	6	18.6
28	176.0		
29	33.3		
30	24.8		
